# The Electrochemical Behavior of Ti in Human Synovial Fluids

**DOI:** 10.3390/ma15051726

**Published:** 2022-02-25

**Authors:** Yueyue Bao, Anna Igual Muñoz, Claes-Olof A. Olsson, Brigitte M. Jolles, Stefano Mischler

**Affiliations:** 1 École Polytechnique Fédérale de Lausanne, CH-1015 Lausanne, Switzerland; anna.igualmunoz@epfl.ch (A.I.M.); claes.o.olsson@gmail.com (C.-O.A.O.); stefano.mischler@epfl.ch (S.M.); 2 Swiss BioMotion Lab, Lausanne University Hospital and University of Lausanne (CHUV-UNIL), CH-1011 Lausanne, Switzerland; brigitte.jolles-haeberli@chuv.ch; 3 Institute of Microengineering, Ecole Polytechnique Fédérale Lausanne (EPFL), CH-1015 Lausanne, Switzerland

**Keywords:** human synovial fluid, electrochemistry, Ti

## Abstract

In this study, we report results of the interaction of titanium (Ti) with human synovial fluids. A wide palette of electrochemical techniques was used, including open circuit potential, potentiodynamic methods, and electrochemical impedance. After the electrochemical testing, selected surfaces were analyzed using Auger Electron Spectroscopy to provide laterally resolved information on surface chemistry. For comparison purposes, similar tests were conducted in a series of simulated body fluids. This study shows that compared to the tested simulated body fluids, synovial liquids show a large patient variability up to one order of magnitude for some crucial electrochemical parameters such as corrosion current density. The electrochemical behavior of Ti exposed to human synovial fluids seems to be controlled by the interaction with organic molecules rather than with reactive oxygen species.

## 1. Introduction

A general requirement of corrosion testing is to select the proper environment. It has to be close to the target environment, and sometimes allows for accelerated testing. Many materials used in the body today are passive materials (CoCrMo, Ti, 316L), and relevant corrosion testing is primordial for assessing materials’ performance.

Metal implants can lead to harmful release of metal into the blood stream [1,2,3,4] by corrosion. This may cause inflammation, making the body environment even more aggressive to the implant [5]. From the chemical aspect, synovial liquid mainly consists of ions such as Na^+^, Mg^2+^, Fe^3+^, K^+^, HPO_4_^2−^, and Ca^2+^ [6]. On a molecular-cell level, serum proteins, lipids, hyaluronic acid, lubricin, and cells are found [7]. The exact composition varies in principle among patients. To somewhat standardize the testing, solutions to simulate human synovial liquid have been designed, including isotonic sodium chloride, phosphate buffers, as well as the more complex Ringer’s and Hank’s solutions. Bovine serum albumin (BSA) or hyaluronic acid is sometimes added to these variants. The relevant test temperature is 37 °C. Attempts to simulate inflammations have been made by the addition of hydrogen peroxide (H_2_O_2_). Previous research has shown that these constituents have a various influence on the corrosion behavior of Ti. For example, BSA can inhibit the reduction reaction by adsorbing onto the metal surfaces [8,9,10,11,12,13]. However, depending on the concentration, protein either can accelerate the corrosion rate of metallic implants by forming soluble complexes with metal ions [11,12,14] or inhibit corrosion by acting as a physical barrier layer [11,13,15]. Similarly, passivation current density slightly decreases with the addition of calcium and phosphate ions due to their adsorption onto metal surface [16,17,18,19]. Besides, a number of studies show that H_2_O_2_ can strongly increase the corrosion rate of implants [20,21,22,23,24]. A summary of ex vivo and in vitro studies has been written by Espallargas et al. [25]. They found a large variability between surfaces, and the organic layers found on implant materials sometimes also appeared as graphite for ex vivo studies. The large variability between patients and surfaces was also confirmed in an in vivo study by Igual-Muñoz et al. [26] following the electrochemistry of a CoCrMo alloy when brought in contact with human synovial liquid. This study showed that in particular the anodic reactivity of CoCrMo was patient dependent and was stronger compared to in vitro experiments.

However, the electrochemical behavior of Ti and its alloy in human synovial fluids has not yet been investigated. Hence, it was chosen to study here the interaction of Ti with synovial liquids extracted directly from the human body for a series of patients at different stages. For this purpose, a multi-electrode set-up is presented specifically designed to perform relevant electrochemical tests. This was complemented with surface analysis by Auger Electron Spectroscopy (AES) to estimate the reactivity of the surface.

## 2. Experimental

### 2.1. Materials and Experimental Set-Up

A Ti rod (grade 2: Ti ≥ 98.9, Fe ≤ 0.30, O ≤ 0.25, C ≤ 0.08) supplied by Goodfellow was used in this study. Samples with 4 mm in diameter and 6 mm in thickness were cut from the rod. The samples were manually polished with SiC emery paper of 1200, 2400, and 4000 grit in water. In order to get a mirror-like surface, final polishing was carried out on a polish tissue with an ethanol-based suspension dispersed with diamond particles (0.25 μm in diameter). The final roughness, as measured by a laser scanning confocal microscope (KEYENCE VK-X200), was Ra 15 ± 3 nm. After that, the samples were cleaned in the ultrasonic bath with acetone for 5 min and subsequently sterilized in 70% ethanol for 5 min and then dried with oil-free compressed air.

**Figure 1:**
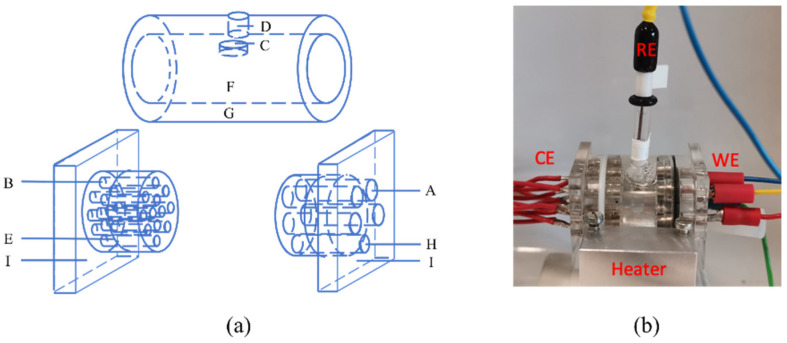
(**a**) Schematic diagram of the three-electrode cell. A: working electrode, B: counter electrode, C: reference electrode, D: air evacuation hole, E: sealing O-ring, F: tested synovial fluid, G: vessel, H: injection hole, I: vessel cover. (**b**) The picture of the cell.

A multi-electrode cell with 2 mL volume was designed to meet the small amount of synovial fluid (normally around 2–7 mL) extracted from patients. The diagram as well as the picture of the cell is presented in Figure 1. For each test, two Ti samples were placed in different positions to test the electrochemical reactions. Stainless steel samples were inserted in the two remaining working electrode slots but for the sake of clarity, the measurements obtained on these electrodes are not discussed here. In one patient only (P17), four Ti samples were measured to check the repeatability of the electrochemical behavior of Ti in a given synovial liquid, as is shown in the results section. The cell included an Ag/AgCl (3.5 M KCl) reference electrode (RE) and 12 counter electrodes (platinum rods) evenly distributed in the vessel cover. The potential of the reference electrode is 0.198 V relative to the standard hydrogen electrode. In order to check the homogeneity of synovial fluids, the OCP of each counter electrode was measured at the beginning of the immersion. After that, all the platinum (Pt) rods were short circuited to serve as the counter electrode (CE). Prior to any experiment, the CE was polished using diamond paste and subsequently cleaned and sterilized in 70% ethanol. The RE electrodes were cleaned and also sterilized in 70% ethanol before and after experiments.

The synovial fluid was extracted and collected with a syringe, and it was directly transferred from the surgery room to the electrochemical laboratory within 5 min. The picture of synovial fluid in the syringe was taken right after transfer from the surgery room. The pH of the fluid was then measured with pH paper (sensitivity is 0.3 pH units) by depositing a drop from the syringe onto the paper. Subsequently, synovial fluid was injected into the cell through the injection hole until complete filling. This operation lasted less than 10 s. A sufficient amount of liquid could be extracted only from certain patients. Over 18 patients, 2 patients yielded not enough liquid (P16 and P18), while in other patients no liquid could be found (P7, P9, P12). Liquids from patients P1, P8 and P10 were used for other experiments. The injection and evacuation holes were closed with silicone caps right after injection to avoid contamination from the air. The cell was placed in an aluminum block to maintain the temperature at 37 ± 1 °C. The whole set-up was installed in a Faraday cage to avoid electrostatic interferences.

### 2.2. Electrochemical Experiments

**Figure 2:**
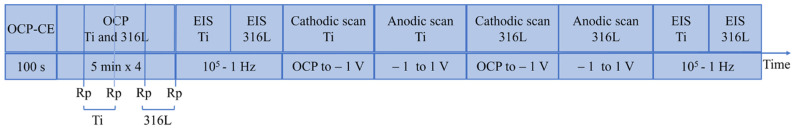
Experimental sequence.

Different electrochemical measurements were carried out sequentially on the samples with an Ivium potentiostat. (a) Open circuit potential (OCP) between each Pt rod and RE was measured in sequence every 10 s, and the whole measurement lasted for 100 s; (b) OCP between working electrodes and the reference electrode was continuously recorded for 20 min. The measurement consists of four loops, and different samples were tested in each loop; (c) Polarization resistance (Rp) of one Ti and one 316L sample was measured at the end of each OCP loop by scanning the potential from −20 to 20 mV with respect to OCP with a scan rate of 2 mV/s; (d) Electrochemical impedance spectroscopy (EIS) was tested on the same Ti and 316L sample at the OCP. The applied potential amplitude was ±10 mV and the frequencies ranged from 105 to 1 Hz; (e) Potentiodynamic scan was performed on the same Ti and 316L sample by scanning the potential from the OCP towards the cathodic direction to −1 VAg/AgCl and reversing towards anodic direction up to 1 VAg/AgCl with a scan rate of 2 mV/s. (f) EIS was conducted on Ti and 316L samples after the polarization scan. After the electrochemical experiments, the pH of tested synovial fluids was measured, and the samples were cleaned in an ultrasonic bath with 70% ethanol for 2 min and blow-dried with oil-free compressed air. The whole experiment procedure is shown in Figure 2. 

For comparison purposes, the electrochemical behavior of Ti was also tested in simulated body fluids (composition is given in Table 1) using the same procedure and conditions applied for synovial fluids. NaCl solutions are the simplest fluid used for simulating body fluids. Incorporation of bovine serum albumin (BSA) is widely used to assess the influence of the organic molecules contained in body fluids [9,10,11,12,13]. H_2_O_2_ was added into simulated fluid to investigate the influence of reactive oxygen species (ROS) [22,23,24]. The solutions were prepared using reagents: NaCl (Sigma-Aldrich, Gribskov, Denmark), BSA (Fisher Scientific AG, Basel, Switzerland), and H_2_O_2_ (Reactolab S.A., Servion, Switzerland). The pH was adjusted to 7.4 by using small amounts of NaOH.

The small volume of the cell may introduce artefacts due to significant modification of the chemistry of the electrolyte, possibly introduced by the polarization. For example, hydrogen and oxygen generated at the counter electrode during anodic or cathodic polarization may diffuse in the small cell towards the working electrodes, thus disturbing the electrochemical measurement. Moreover, pH changes due to electrolysis may have a significant effect because of the small volume of the cell. In order to check for such possible artefacts, the small cell was validated by comparison with a standard size corrosion cell. The results are shown in the supplementary materials file (Figures S1–S3, Table S1) attached to this paper. The results show that no obvious artefacts occurred in this small volume cell used here.

### 2.3. Human Synovial Fluids Sampling

Three kinds of patients were selected, and their synovial fluids were extracted by a skilled surgeon in the surgery room:

- Group 1, Primary surgery patients (PS): patients who do not have an implant but exhibit different grades of knee inflammation and clinical states.
- Group 2, Total Knee Arthroplasty (TKA): patients with a Total Knee Arthroplasty implant.
- Group 3, Revisions (R): patients who will go for revision surgery due to a failure, rejection, or problem with the implant.

Synovial fluids were extracted during surgery (group 1 and 3) and puncture (group 2). All care was taken by the surgeon to avoid contamination of the synovial fluid by blood or other components. The extraction procedure is explained in detail in [26]. The overall protocol of this study (protocol 208/13) was approved on 28 May 2013 by the ethics committee for human being studies of the local government (Commission cantonale (VD) d’éthique de la recherché sur l’être humain) according to the ICH GCP guidelines.

### 2.4. AES Analysis

The surface composition and film thickness analysis of the non-polarized Ti samples of P4 and P5 were performed using a PHI 680 scanning Auger microscope (Physical Electronics, Eden Prairie, USA) equipped with a scanning electron microscope and an argon ion gun. Before the analysis, the samples were stored in the desiccator for at least 2 weeks. The measurements were done at a voltage of 10 kV and a current of 10 nA for the electron gun and a voltage of 1 kV and 500 nA for the ion gun with a raster size of 2 × 2 mm. The analysis was conducted with the samples tilted at 30 degrees with respect to the incident electron beam and 45 degrees with respect to the incident ion beam. Under these conditions, the sputter rate of TiO_2_ is 0.6 nm/min (2 nm/min on SiO_2_).

## 3. Results

### 3.1. Human Synovial Fluids

**Figure 3:**
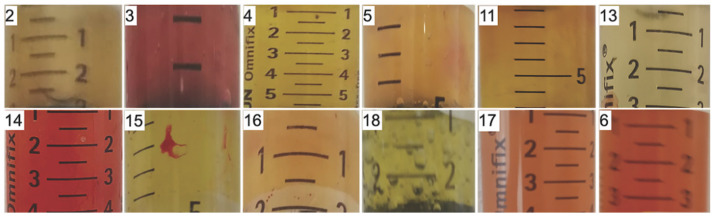
Pictures of human synovial fluids inside the syringe before electrochemical measurements.

The information of tested synovia is listed in Table 2 and the pictures are shown in Figure 3. The results show that the pH of most extracted synovia is around 7.4, except for the fluids from patient 6 (P6), 13, and 16, which are very slightly more acidic or alkaline, respectively. No significant difference in pH value of the synovial fluids was observed at the end of the experiment. As shown in Figure 3, the color of the synovial liquids varied depending on patient, with no obvious correlation. The OCP of the twelve Pt wires used as counter electrodes was used for assessing the degree of homogeneity of the liquid at the mm scale. Note that the OCP of electrodes is subject to variations depending on details in sample preparation (polishing, cleaning). To take this into account, the standard deviation of OCP of the Pt electrodes was used as an indicator. For reference, the standard deviation value measured in 0.8% NaCl solution, taken here as a reference for a homogeneous solution, was 32 mV. This value varied depending on the patient between a minimum of 20 mV and a maximum of 65 mV. These values are very close to what is found in the NaCl reference solution, and it may, therefore, be concluded that the synovial liquids are homogeneous, at least at the mm scale.

### 3.2. OCP of Ti in Synovial Fluids

**Figure 4:**
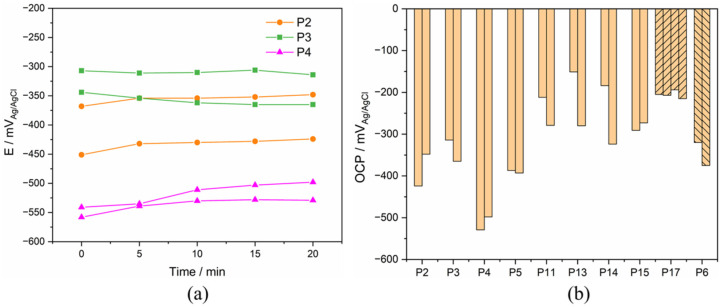
(**a**) Evolution of OCP with immersion time in the synovia from P2–4 and (**b**) stabilized OCP results of Ti after 20 minutes’ immersion in different synovial fluids from PS, TKA (P17), and R (P6) group.

The OCP of the Ti electrodes was measured in sequence every 5 min. An example of OCP evolution with time for P2, P3, and P4 is displayed in Figure 4. The OCP value slightly varies during immersion time (variation less than 2.5 mV/min). Interestingly, it can either increase with time (for example patients 2 and 4) or decrease (Patient 3). Similar trends of OCP were also observed in other tested synovial fluids.

The comparison of the stabilized OCP values measured after 20 minutes’ immersion in all synovial fluids is presented in Figure 4. The stabilized OCP varies among patients between a maximum of −151 mV_Ag/AgCl_ and a minimum of −529 mV_Ag/AgCl_. In some cases (P11, P13, and P14), the two Ti electrodes immersed in the same synovial fluid differ significantly in their OCP value (difference larger than 50 mV). This can be due to slight, uncontrolled differences in sample preparation or contamination during sample storage before the experiment.

### 3.3. Polarization Resistance

**Figure 5:**
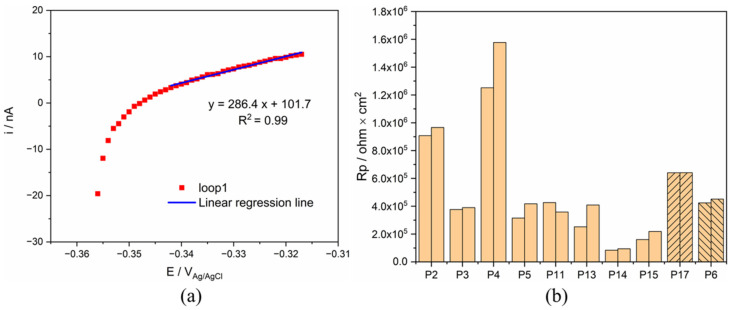
(**a**) Rp measurement of Ti for P6 and (**b**) comparison of Rp value of Ti in all synovial fluids.

Polarization resistance (Rp) of both Ti was conducted during OCP measurement, and the result of Ti in synovia from patient 6 was displayed in Figure 5 as an example. A linear relationship between current and potential was observed only in the anodic current domain. Rp was determined as the reciprocal of the slope of the linear part section, as shown in Figure 5. The obtained values for all patients are also plotted in Figure 5. These results indicate that Rp varies significantly with patients, P4 being the highest and P14 the lowest. Note that the polarization resistance response differs from an ideal behavior (linear variation of current across the zero current point) where the current is determined only by the charge transfer reaction kinetics [27]. Only in this ideal case, the reciprocal of the resistance is proportional to the corrosion current density. This is not the case in the present measurements where a linear behavior is only observed in the anodic domain. To interpret the present results, other effects must be considered, such as capacitive effects potentially induced by the presence of the passive film and adsorbed organic molecules. These points will be discussed in Section 4.3.

### 3.4. Solution Resistance

**Figure 6:**
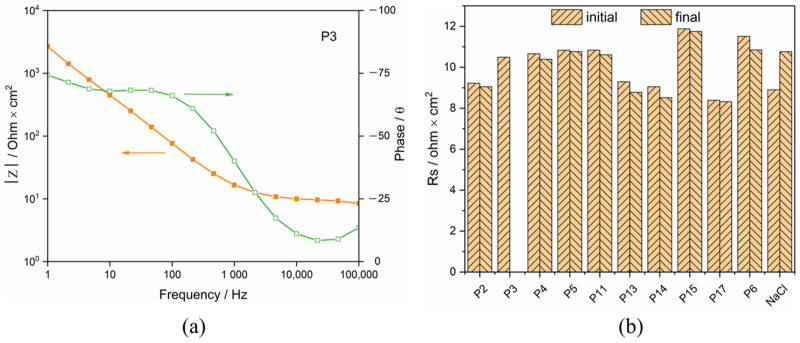
(**a**) Bode plot of Ti in synovia from P3 and (**b**) Rs of synovial fluids from different patients (initial: before potentiodynamic polarization, final: after all measurements completed).

The electrical resistance of the synovial fluids was extracted from the impedance spectra measured before potentiodynamic polarization and after all measurements were completed, and this is for checking possible changes of the synovial fluids induced by the polarization. A typical Bode plot is presented in Figure 6. The resistance at a frequency of 10^5^ Hz was considered as representative of the ohmic resistance of the synovial fluids. They are plotted in Figure 6 for the different patients and, as a reference, for the 0.8% NaCl solution. For P3 and P17, the software suddenly broke down, thus the solution resistance of synovial fluid was not measured at the end of the measurement. Clearly, synovial fluids are conductive and present a resistance similar to a simple 0.8% NaCl solution. Moreover, no significant difference in the solution resistance tested before and after PD scan was obtained.

### 3.5. Potentiodynamic Polarization Curve

**Figure 7:**
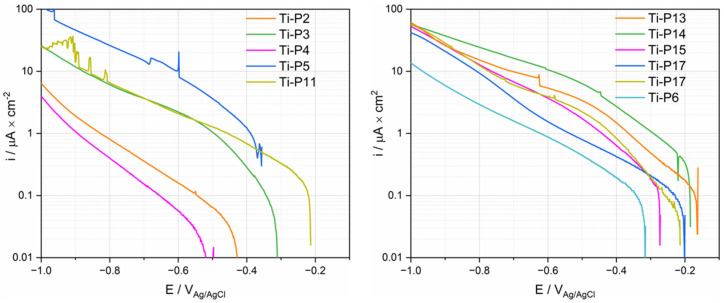
Cathodic polarization curves (logarithmic scale of the absolute current density) of Ti tested in synovial fluids from PS, TKA (P17) and R (P6) group.

Potentiodynamic polarization was measured by first cathodically polarizing the samples and reversing the scan to the anodic direction when a potential of −1 V_Ag/Ag/Cl_ was reached. Figure 7 shows the measured cathodic scans. The current density varies significantly depending on the patients. Particularly low current densities are found in the case of P2 and P4. All curves except P5 exhibit a linear part approximately 100–200 mV below the OCP. P13–17 exhibit a second linear part at higher polarization with a slightly different slope. This indicates that the cathodic kinetics is under charge transfer control and could involve the reduction of oxygen, protons and/or water. 

**Figure 8:**
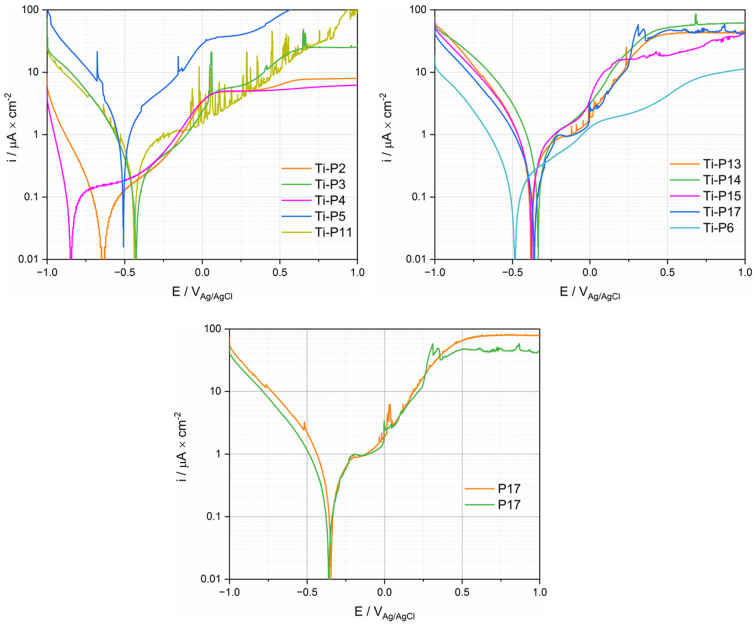
Anodic polarization curves (logarithmic scale of the absolute current density) of Ti tested in synovial fluids from PS, TKA (P17 with two repetitions), and R (P6) group.

Anodic polarization curves of Ti for the PS group are displayed in Figure 8. Three reaction domains were observed in polarization curves: the cathodic domain below the corrosion potential (E_cor_) where the current density is mainly determined by the reduction reaction, the cathodic/anodic transition at E_cor_ and, at higher potentials, the anodic domain. This domain is typically characterized by an initial steady increase in current followed by a plateau. Only P11 does not show any plateau in the investigated potential range. The anodic branch of the polarization curves of P11 and P17 exhibit a sudden current oscillation, for which the origin is not clear. Interestingly, there is a certain proportionality between cathodic and anodic current densities, i.e., patients exhibiting larger anodic current also exhibit larger cathodic current densities. This suggests that both cathodic and anodic kinetics are affected by common factors. Interestingly, the two polarization curves measured on two Ti samples in the same fluid from P17 exhibit very similar behavior in the anodic domain (Figure 8), while in the cathodic domain (Figure 7 and Figure 8), some small differences appear between the two measurements. This good repeatability confirms the pertinence of electrochemical measurements in synovial fluids. Not surprisingly, Figure 8 also exhibits large differences in results among patients, as already observed in the cathodic polarization curves.

In order to quantitatively assess this scatter, characteristic parameters such as OCP, the cathodic current density i_c_ measured at a potential of −0.9 V_Ag/AgCl_ in the anodic scan, the corrosion potential E_cor,_ and the anodic current density i_pp_ measured in the passive range at a potential of 0.5 V_Ag/AgCl_ were extracted from Figure 8 and listed in Table 3. The E_cor_ value ranges between a maximum of −0.334 V_Ag/AgCl_ up to a minimum of −0.636 V_Ag/AgCl_. The cathodic current densities listed in Table 3 may vary among patients by two orders of magnitude while the anodic values exhibit less variation (one order of magnitude).

Note that in the Table 3, OCP values differ from Ecor values. This is not surprising, since they are obtained under different experimental conditions. OCP is measured in absence of any imposed external current. The Ecor corresponds to the potential in the polarization curves when the current changes the sign, typically at the cathodic to anodic transition. Thus, Ecor depends on experimental parameters, such as scan rate, current sensitivity, and feedback loop of the potentiostat, that do not affect OCP measurement. From an electrochemical point of view, OCP is the potential spontaneously attained by the electrode immersed in the solution. Note that OCP may change with time due to possible variation of electrolyte, electrode surface and interface (double layer). During measurement of Ecor, the electrode has already experienced polarization typically at cathodic potential, changing the original electrode state established at OCP. For example, cathodic polarization may modify the passive film and/or adsorbed layers. Thus, during measurement of Ecor, the electrode is experiencing the dynamic situation imposed by the potentiostat and the associated test parameters. This electrochemical situation is very different from the OCP measurement conditions. The difference between OCP, Ecor, and other potentials is extensively discussed in the reference [28].

### 3.6. Surface Analysis

**Figure 9:**
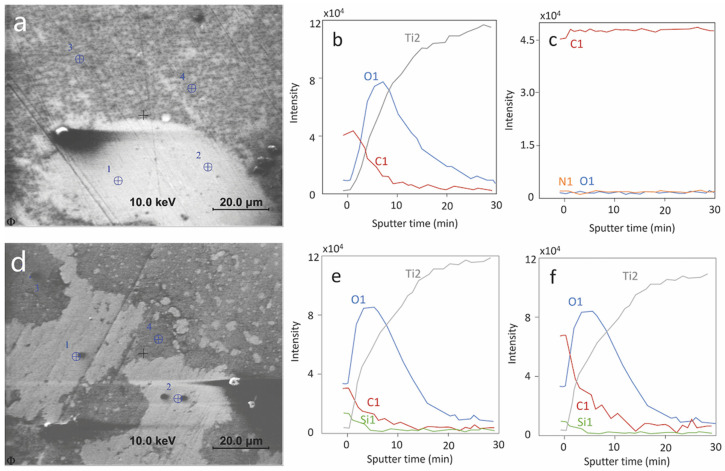
AES analysis of Ti surfaces exposed at OCP to synovial fluid from patient 4 and 5. (**a**–**c**) are for P4; (**d**–**f**) are for P5; (**a**,**d**): secondary electron images and analyzed points; (**b**,**e**): depth profile on analysis point 1; (**c**,**f**): depth profile on analysis point 3; Differential intensity is plotted in the depth profile.

AES analysis was carried out on samples exposed for a total duration of 1.5 h at OCP to the synovial fluids of patients P4 and P5, patients that showed the lowest and highest current densities in the polarization curves (Figure 8). Figure 9 represents the secondary electron images (a,d) of the analyzed surface with the AES measurement points marked 1 to 4. The depth profiles measured on points 1 and 3 of each sample are also shown. The measurement on points 2 and 4 yielded similar results as points 1 and 3. The P5 surface is extensively covered with a deposit appearing dark grey in Figure 9a. According to the AES depth profiles (Figure 9c) this layer is thicker than approximately 30 nm and is mainly composed of carbon with small amounts of nitrogen and oxygen. It is likely to be formed by adsorption or precipitation of organic molecules from the synovial fluids. Note that this precipitation can occur during immersion in the synovial fluid or during cleaning with the alcohol/water solution. On P5 this relatively thick carbonaceous layer is not observed and, instead, a thin carbon layer of thickness of a few nanometers thickness (varying between locations) appears. Similar carbon contamination is observed in the locations of P4 where the thick carbonaceous layer was absent. The oxide film formed on Ti is very similar in both patients as would be expected for a passive film formed during exposure at similar potentials as the case for both P4 and P5. Interestingly, P5 shows surface contamination by Si, which is an element reported to appear in blood plasma [29] and synovial fluid [30].

### 3.7. Electrochemical Results for Simulated Fluids

**Figure 10:**
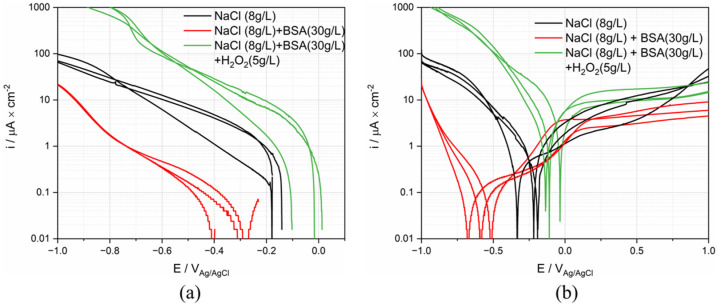
(**a**) Cathodic and (**b**) anodic polarization curves (logarithmic scale of the absolute current density) of Ti in simulated body fluid solutions.

The electrochemical behavior of Ti was tested in the three different simulating body fluids listed in Table 1. Each measurement was repeated 3 times. The representative polarization curves are presented in Figure 10. From this Figure, it appears that the solution composition mainly affects the cathodic reactivity (differences in cathodic current up to two orders of magnitude) while the anodic behavior is less affected (current densities within the same order of magnitude). As previously reported, albumin acts as an inhibitor of the oxygen reduction reaction [9,10,11,12,13]. This explains the significant lowering of the cathodic current density observed in the BSA solution. The increase of current density in the cathodic domain by adding H_2_O_2_ is expected because of the strong contribution of H_2_O_2_ reduction. The slightly enhanced corrosion in the anodic domain could be attributed to the formation of thicker and porous passive film because of the large amount of OH^−^, which leads to the release of Ti [22,31,32,33]. Contrary to the cathodic domain, the BSA slightly lowers the anodic current density.

The same parameters extracted from ex vivo, were extracted from cathodic and anodic polarization curves and summarized in Table 4. The standard deviation (STDEV) and error were calculated to assess the reproducibility of the polarization curve. As is shown in the table, the corrosion behavior of Ti in BSA containing solution shows the best reproducibility.

## 4. Discussion

### 4.1. Electrochemical Reactions of Ti in Synovial Fluids

As shown in Figure 7 and Figure 8, the electrochemical behavior of Ti is patient dependent, especially for the cathodic reaction. The cathodic current is given in principle by the reduction of water, dissolved oxygen, and protons. However, the contribution of proton reduction is estimated to be lower than 0.1 µA/cm^2^ at pH 7 [34], thus proton reduction can be disregarded in the following discussion. The presence of dissolved molecular oxygen in the synovial fluid was reported by Lund-Olesen, who measured partial pressures in the range 20–87 mm Hg depending on the clinical state of the patient [35]. For comparison, the partial pressure of oxygen in the air is 160 mm Hg, corresponding to an equilibrium concentration of dissolved oxygen in the water of 8 mg/L at 25 °C. So, the oxygen reduction reactions are expected to significantly contribute to the electrochemical behavior in synovial fluids. The electrochemical reduction kinetics of dissolved molecular oxygen is controlled by a number of phenomena, such as the charge transfer at the electrode-solution interface and the mass transport of oxygen from the bulk solution to the interface [27]. Moreover, in body fluids, adsorption of organic molecules may also affect the oxygen reduction by acting as a barrier preventing oxygen from reaching the electrode surface [12,15]. As is shown in AES depth analysis results, various organic adsorption was observed on the sample surface, depending on the patients. In conclusion, the oxygen contribution to the reduction kinetics depends on its concentration and the concentration of adsorbing organic species and can thus vary among patients depending on their clinical state. This explains the large scatter in the cathodic kinetics observed in the present study.

Corrosion studies of Ti in simulated fluids have shown that the passive film of Ti mainly consists of Ti_2_O_3_ and TiO_2__,_ with the TiO_2_ concentration increasing with potential [36,37]. The passive current density is determined by the rate of ion transportation through the passive film and the stability of the film against dissolution. TiO_2_ is thermodynamically stable in the pH ranging from 2 to 12, and only dissolves in the presence of specific species, such as HF and concentrated H_2_SO_4_ [38], thus the influence of pH on anodic reaction can be negligible. This deduction can be confirmed by the results in the literature that the anodic current density varies little with pH from 2 to 7 [39,40]. The variation of passive current density was observed to depend on the protein concentration [11,13,14,38]. At low concentration, protein can interact with metal ions by forming soluble complexes, thus increasing the dissolution rate of metal, while at high concentration its adsorption inhibits the corrosion [11]. Dissolved ions may also influence the passive current of Ti. For example, it was reported that Ca ions reduce the cathodic current of Ti by adsorbing onto metal surface [12,18], while it has little influence on anodic corrosion behavior [12]. The presence of phosphate contributes to the formation of a protective layer and thus increases the corrosion resistance of Ti [18,41]. Silicates inhibit the corrosion of Ti in alkaline hydrogen peroxide solutions [42]. According to the AES analysis, the difference in anodic and cathodic current density seems to be determined by the formation of the organic layer on the Ti surface.

### 4.2. Corrosion Current Density

The corrosion current density i_cor_ can be obtained by extrapolating the linear part of the logarithmic plots illustrated in Figure 7 to the OCP potential. Depending on the patient, the i_cor_ values range between the extremes of 0.025 (P4) and 2 (P5) μA/cm^2^ with however most of the patients lie in the narrower range 0.1 to 0.5 μA/cm^2^. Corrosion current densities of Ti measured in body simulated fluids span in a very similar range from 0.02 to 0.6 μA/cm^2^, but relatively high corrosion current densities observed in P5 were never reported [12,43].

In order to compare the corrosion rate with real conditions, the corrosion current density (A m^−2^) can be converted to mass loss m_cor_ (mg dm^−2^ day^−1^) through Faraday’s law with the equation as below:

(1) $$m_{\mathrm{cor}}={\mathrm{Mi}}_{\mathrm{cor}}/\left(8640\;\mathrm{nF}\right)$$

where M is the atomic mass of Ti: 48 g mol^−1^, F is Faraday constant: 96,485 A s mol^−1^, and n is its oxidation valence which is assumed to be 4. The mass loss obtained from the equation varies depending on the patients from a minimum of 0.06 to a maximum of 2.58 mg dm^−2^ day^−1^.

While concentrations of metallic ions in serum, plasma, whole blood, and urine are largely reported in the literature, no information on actual in vivo corrosion rates of titanium can be found. The corrosion rate can be tentatively estimated by considering that, in case of no further ion accumulation in the body, it corresponds to the release rate of titanium through urine. This is a coarse assumption, as ions can be eliminated through other ways, such as sweat and hair growth [3]. The typical urine release rate of human bodies is approximately 1.5 l per day. Titanium concentration in urine was reported by Matusiewicz [2] to range from 2 × 10^−4^ mg/L to 0.65 mg/L for hip and knee titanium bearing implants. Considering an approximative titanium exposed implant surface of 1 dm^2^ this yields an elimination rate of Ti through urine in the range 0.003 to 1 mg dm^−2^ day^−1^. This range corresponds well to the corrosion rates observed here. This suggests that corrosion is an important factor responsible for the release of Ti ions from metallic implants.

### 4.3. Comparison with Tests in Simulated Fluids

**Figure 11:**
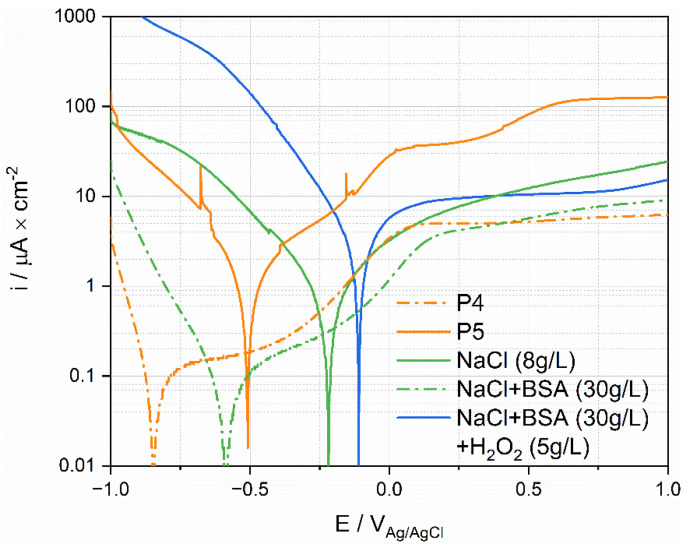
Anodic polarization curves of Ti tested in simulated fluids and in synovial fluids.

Representative polarization curves measured in the different solutions were plotted in Figure 11, together with the polarization curves measured for P4 and P5. These patients define the envelope of all the curves measured on patients (see Figure 8). Interestingly the cathodic part of the curve measured in the NaCl solution lies outside the envelope of the ex vivo measurements. Adding BSA moves the polarization curve (cathodic part) into the ex vivo domain. The addition of H_2_O_2_ generates very high cathodic currents. This is not surprising as hydrogen peroxide is a strong oxidizing agent. This may indicate that the cathodic reactivity of Ti in synovial fluids is mainly determined by the presence of organics species that likely affect the reduction rate, and thus the corrosion rate, by adsorbing on the surface of the metal. In situ measurements of the extent of adsorption, for example using EQCM, should be carried out to confirm this hypothesis.

Interestingly, the anodic domain is less affected by the nature of the environment than the cathodic one. The results for simulated fluids do not allow to reproduce the high anodic current densities observed in P5, even when adding a strong oxidizing agent. This indicates that the oxidative strength of the solution is not necessarily the key parameter. Possibly, the large anodic currents obtained in P5 are due to specific molecules in synovial fluid, such as protein [11,14], that promote passive film dissolution.

**Figure 12:**
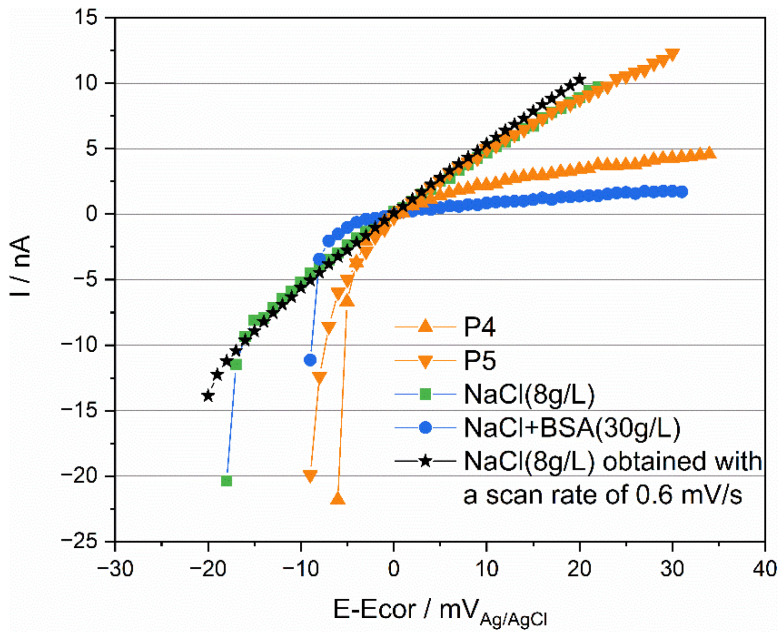
Rp measurements of Ti tested in simulated fluids and in synovial fluids with a scan rate of 2 mV/s and 0.6 mV/s.

The peculiar behavior observed in Figure 5 during the measurement of polarization resistance can also be attributed to an effect of protein. Figure 12 compares the obtained results for P4 and P5 as well as the corresponding results obtained in 0.8% NaCl and in 0.8% with 30 g/L BSA. For the sake of clarity, the polarization (E-E_cor_) was reported in the abscissa instead of the actual electrode potential E. In the simple NaCl inorganic solution, a well-defined linear behavior crossing the zero current is observed. Deviations from linearity are observed at the onset of polarization at the cathodic potential. This is likely related to the capacitive effects due to double layer and passive film charging. Indeed, tests carried out at a lower scan rate (0.6 mV/s) showed an identical behavior but with a much smaller deviation from linearity at the onset of polarization. Interestingly, the response of P5 is quite close to the behavior observed for NaCl solution (same slope in the anodic domain) with, however, a larger deviation from linearity in the cathodic domain possibly due to differences in open circuit potential (nearly 200 mV) and the associated differences in the double layer structure, adsorption, and passive film properties. Compared to P5, P4 shows a higher polarization resistance (reciprocal of the slope in the anodic domain). Interestingly, the same trend is found when adding BSA to the NaCl solution. This further supports the interpretation of Figure 11, indicating that the organic substances play a key role in the electrochemical response of titanium exposed to synovial fluids.

## 5. Conclusions

This study is the first systematic investigation of the electrochemical response of Ti exposed to human synovial fluids extracted from various patients. It leads to following conclusions:

- The electrochemical response of Ti in both cathodic and anodic domains was found to significantly (several orders of magnitude) vary among patients.

The calculated corrosion rate extracted from the polarization curves varies depending on the patients from a minimum of 0.025 to a maximum of 2 μA/cm^2^ (0.06 to 2.58 mg dm^−2^ day^−1^). These values were found to be consistent with the Ti release rate from hip and knee artificial joints implanted in humans.

- Ex situ AES surface analysis of Ti samples exposed to synovial fluids extracted from two patients revealed a similar passive film thickness but a large difference in the surface coverage by organic species. Interestingly, the polarization curves of Ti in these two synovial fluids were very different. This indicates that organics play a crucial role in the electrochemical responses of Ti exposed to synovial fluids.
- The previous conclusion is supported by the fact that simulated body fluids containing organic molecules such as BSA can better simulate the ex vivo electrochemical behavior of Ti than those containing BSA or oxidizing agents such as H_2_O_2_.

## Figures and Tables

**Table 1:** Composition of the simulated body fluids.

Solution	NaCl	BSA	H_2_O_2_
NaCl	8 g/L	-	-
NaCl + BSA	8 g/L	30 g/L	-
NaCl + BSA + H_2_O_2_	8 g/L	30 g/L	5 g/L

**Table 2:** Properties of human synovial fluid.

Patient	Group	Volume/mL	pH
2	PS	6	7.4
3		2.5	7.4
4		5.5	7.4
5		4	7.7
11		7	7.4
13		>10	7
14		8	7.4
15		5	7.4
16		1.5	7.9
18		1	7.3
17	TKA	11	7.3
6	R	6	6.8

**Table 3:** Electrochemical parameters extracted from the anodic polarization curves of Ti samples.

			Anodic Curve		
Patient		OCP/mV	i_c_ (−0.9 V)/µA/cm^2^	E_cor__/_V_Ag/Ag/Cl_	i_pp_ (0.5 V)/µA/cm^2^
PS	2	−386	−1.2	−0.636	6.29
	3	−340	−13.3	−0.425	16.41
	4	−514	−0.2	−0.843	5.23
	5	−391	−31.8	−0.508	81.91
	11	−246	−10.3	−0.439	10.46
	13	−216	−34.6	−0.378	40.91
	14	−254	−35.1	−0.334	52.16
	15	−282	−26.8	−0.377	18.47
TKA	17	−201	−25.8	−0.346	47.06
R	6	−348	−6.7	−0.487	4.22
Average		−318	−19.35	−0.46	31.13
STDEV		101	12.62	0.15	24.46
Error		0.6	0.89	0.56	1.23

**Table 4:** Electrochemical parameters extracted from the anodic polarization curves.

Solution		OCP/mV	Anodic Curve		
			i_c_ (−0.9 V)/μA/cm^2^	E_cor_/ V_Ag/Ag/Cl_	i_pp_(0.5 V)/μA/cm^2^
NaCl (8 g/L)	Average	−201	−46.70	−0.3	7.9
	STDEV	61	17.65	0.06	3.39
	Error	0.36	0.49	0.27	0.54
NaCl + BSA (30 g/L)	Average	−357	−3.68	−0.59	4.46
	STDEV	47	0.35	0.08	1.31
	Error	0.13	0.09	0.14	0.29
NaCl + BSA + H_2_O_2_ (5 g/L)	Average	−62	−968	−0.09	12.44
	STDEV	67	94.39	0.05	4.17
	Error	1	0.10	0.54	0.31

## Data Availability

The data cannot be made available for patient privacy.

## Supplementary Materials

The following supporting information can be downloaded at: https://www.mdpi.com/article/10.3390/ma15051726/s1, Figure S1: (a) The pictures of the new small cell and (b) typical big cell; Figure S2: The polarization curves of Ti in NaCl solution tested in both cells; Figure S3: The polarization curves of two Ti samples in NaCl solution tested sequentially in the small cell; Table S1: Electrochemical parameters extracted from polarization curves.
