# Supplementary material for: The Electrochemical Behavior of Ti in Human Synovial Fluids
Source: Materials (Basel). 2022 Feb 25;15(5):1726. doi: 10.3390/ma15051726 (PMC8910938; doi:10.3390/ma15051726)
Supplement: Supplementary file 1 [file materials-15-01726-s001.zip › materials-1603022-supplementary.pdf]

materials

Validation of the small electrochemical cell

In this supplementary material document, the feasibility of the small cell was investigated by conducting the corrosion behavior of Ti in NaCl (8g/L) solution in both small cell and typical big cell.

1. Experimental

Ti samples were used for both small cell and big cell, and the detailed sample preparation is shown in the paper. The same experimental set-up was used for the small cell, while a Ag/AgCl (3.5 M KCl) and a graphite rod was served as reference electrode and counter electrode respectively in the big cell. The temperature of the big cell was maintained at 37 ± 1 ^{o}C by heating the water in the interlayer of the cell container. The pH of NaCl solution was adjusted to 7.4 with NaOH. The pictures of the new small cell (2 mL) and typical big cell are presented in Fig.1. For each test, one Ti sample was placed in each cell and the test was carried out for three times to guarantee the reproducibility.

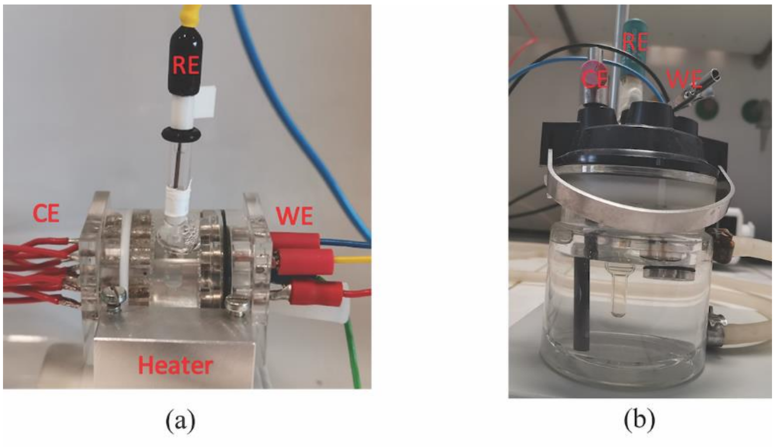

Figure S1. (a) The pictures of the new small cell and (b) typical big cell.

Different electrochemical measurements were conducted sequentially on the Ti sample with a Ivium (small cell) and NOVA potentiostat (big cell) respectively. The experimental procedure is shown as below:

OCP, between Ti and NaCl solution, was continuously recorded for 20 min.

Potentiodynamic scan was performed on the sample by scanning the potential from the OCP towards the cathodic direction to −1 $V_{Ag/AgCl}$ and reversing towards anodic direction up to 1 $V_{Ag/AgCl}$ with a scan rate of 2 mV/s.

2. Results

The polarization curves of Ti in NaCl solution tested in both cells were presented in Fig.2. Results show the polarization curves obtained from small cell are similar with that from big cell, the latter shows better reproducibility. The lower current for small cell at the potential lower than −0.6 $V_{Ag/AgCl}$ might be due to the less amount of H^+ or dissolved O_2 in electrolyte due to the small volume. The higher anodic current density acquired for small cell could also be attributed to the less amount of dissolved O_2, which promotes the generation of passive film thus reduces the passive current density.

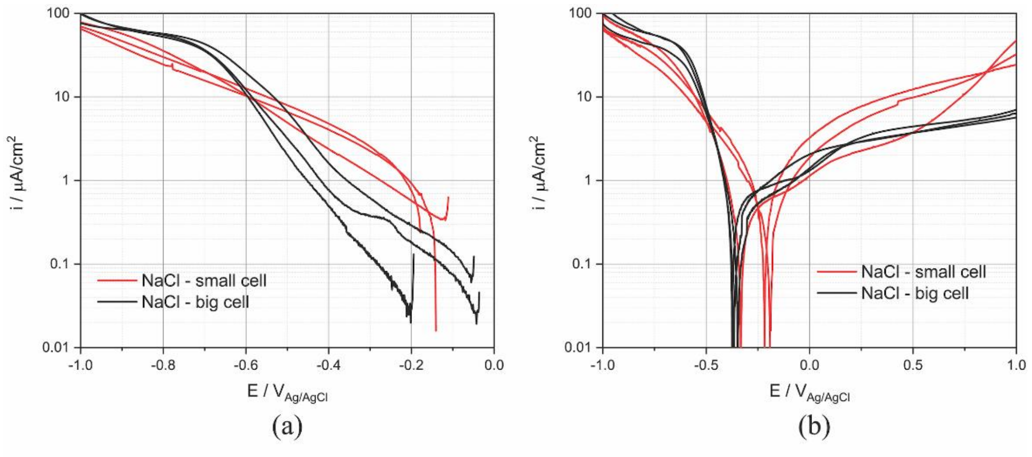

Figure S2. The polarization curves of Ti in NaCl solution tested in both cells.

The electrochemical parameters extracted from polarization curves are presented in table 1. As is shown in the table, no significant difference is obtained for the OCP value of Ti obtained from both cells. Therefore, there is little influence of the cell size on the corrosion behavior of Ti in NaCl solution.

Table S1. Electrochemical parameters extracted from polarization curves.

			Cathodic polarization curve		Anodic polarization curve		
		OCP/ mV	i_c(-0.8V)/ μA/cm^2	i_c (-0.3V)/ μA/cm^2	i_c (-0.8V)/ μA/cm^2	E_{cor}/ mV	i_p(0.5V)/ μA/cm^2
Small cell	Average	-201	-27.64	-1.52	-34.26	-0.30	7.90
	STDEV	61	8.15	0.89	14.66	0.06	0.54
Big cell	Average	-184	-56.60	-0.37	-54.23	-0.36	4.13
	STDEV	60	3.99	0.25	7.35	0.01	0.51

Considering the possible effect of hydrogen and oxygen generated on Pt on the corrosion behavior of Ti, two Ti samples were putted in the small cell and the polarization scan was conducted on both samples in sequence after 20 mins' immersion in NaCl solution. The experiment was conducted three times, and the results are displayed in Fig.3. The identical results for both samples indicate the hydrogen and oxygen generated on Pt has no effect on the corrosion behavior of Ti in NaCl solution.

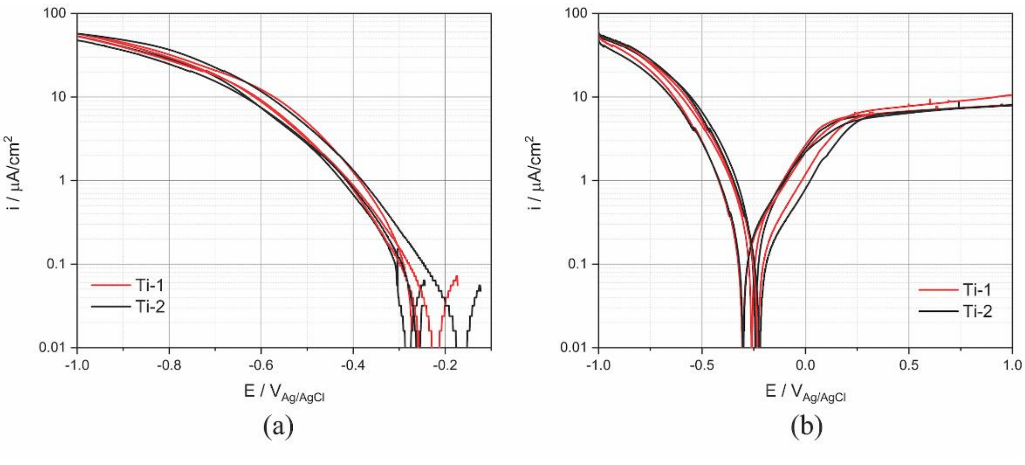

Figure S3. The polarization curves of two Ti samples in NaCl solution tested sequentially in the small cell.

3. Conclusion

Based on the results, conclusion can be drawn as below:

Electrochemical experiments can be conducted by the new small cell with high reliable results based on the similar results with that from the big typical cell.

There is no influence of hydrogen and oxygen generated on Pt on the corrosion behavior of Ti tested in small cell.
